# Metabolic syndrome and breast cancer survivors: a follow-up analysis after completion of chemotherapy

**DOI:** 10.1186/s13098-022-00807-y

**Published:** 2022-03-03

**Authors:** Christina M. Dieli-Conwright, Louise Wong, Sarah Waliany, Joanne E. Mortimer

**Affiliations:** 1 Division of Population Sciences, Dana-Farber Cancer Institute, Harvard Medical School, 375 Longwood Avenue, Boston, MA 02215 USA; 2 Division of Medical Oncology and Experimental Therapeutics, City of Hope Comprehensive Cancer Center, Duarte, CA USA; 3 Department of Medicine, Stanford University School of Medicine, Stanford, CA USA

**Keywords:** Metabolic syndrome, Breast cancer, Body weight, Biomarkers

## Abstract

**Background:**

We previously reported that (neo)adjuvant chemotherapy adversely altered metabolic syndrome (MetS) components, body composition, and related biomarkers after a 12 to 18-week chemotherapy treatment course in women. Here, we sought to determine whether these measures worsened within 4–5 years post-chemotherapy among the same sample of early stage breast cancer survivors.

**Methods:**

Twenty-eight breast cancer survivors were reassessed within 4–5 years post-chemotherapy. Participants were tested for MetS, lipid profile (total cholesterol; TC, low-density lipoprotein cholesterol; LDL-C), glucose metabolism (insulin, homeostatic model- insulin resistance; HOMA-IR, glycosylated hemoglobin; HbA1c), inflammation (C-reactive protein; CRP) and body composition (body weight; BW, percent body fat; BF, fat mass; FM) during follow-up physical exams. A comparison of measurements between post-chemotherapy and follow-up periods was performed using repeated measures analysis of covariance.

**Results:**

Most study patients were Caucasian (44%) or Hispanic (30%) with a mean age of 48.2 years. Average time from completion of chemotherapy was 4.75 years. At follow-up, MetS components significantly increased (p < 0.01) compared with the post chemotherapy assessment. Additionally, BF, FM, lipids (TC, LDL), glucose metabolism (HOMA-IR, insulin, HbA1c), and inflammation (CRP) significantly increased (p < 0.01). Notably BW significantly increased; mean weight gain after chemotherapy was 6.1 kg and increased an additional 8.2% at follow-up (p < 0.01).

**Conclusion:**

MetS components, body composition, and biomarkers continued to worsen within 4–5 years post-chemotherapy in breast cancer survivors. Energy balance interventions should target breast cancer patients to reduce the exacerbation of MetS.

## Introduction

Advancements in screening and (neo)adjuvant therapies are attributed with improvements in breast cancer survival [1]. Breast cancer survivors undergo an onslaught of adverse treatment-associated side effects, such as gains in body weight [2, 3], reductions in physical activity [4], and deteriorating metabolic profiles leading to an elevated risk of cardiovascular disease [5, 6] and metabolic syndrome (MetS) [7, 8]. MetS is a collection of clinical features including hypertension, dyslipidemia, hyperglycemia, and abdominal obesity [9]. MetS is a vastly prevalent disorder, impacting roughly 25% of adults [10], and is associated with a two-fold higher risk for cardiovascular disease and diabetes [11]. A person is diagnosed with MetS when any three of the five following components are present (Table 1): (a) waist circumference (WC) ≥ 88 cm (35 in.); (b) elevated triglycerides (TRI) ≥ 150 mg/dL or on drug treatment for elevated TRI; (c) reduced high-density lipoprotein cholesterol (HDL-C) < 40 mg/dL or on drug treatment for low HDL-C; (d) ≥ 130 mmHg systolic blood pressure or ≥ 85 mm Hg diastolic blood pressure or on antihypertensive treatment; (e) elevated fasting blood glucose (BG) ≥ 100 mg/dL or on drug treatment for elevated glucose [12]. MetS and associated biomarkers, including excess body weight, physical inactivity, insulin resistance, inflammation, and disrupted adipokines, are associated with increased risks of breast cancer, and subsequently all-cause mortality and increased risk of breast recurrence in those diagnosed with breast cancer [13, 14].

**Table 1:** Criteria for the clinical diagnosis of metabolic syndrome

Measure	Categorical cutpoints
Elevated waist circumference	≥ 88 cm (35 in.)
Elevated triglycerides	≥ 150 mg/dL or on drug treatment for elevated triglycerides
Reduced HDL-C	< 40 mg/dL or on drug treatment for low HDL-C
Elevated blood pressure	≥ 130 mmHg systolic blood pressure or ≥ 85 mm Hg diastolic blood pressure or on antihypertensive treatment
Elevated fasting glucose	≥ 100 mg/dL or on drug treatment for elevated glucose

*HDL-C* high-density lipoprotein-cholesterol

MetS features worsen in breast cancer patients following chemotherapy, increasing their risk of death from cardiovascular and metabolic diseases [15, 16]. In fact, a previous report demonstrated that older women (> 70 years of age) who had recently concluded treatments for breast cancer experienced weight gain (51% of cases), hypertension (34%), peripheral vascular disease (26%), and diabetes (13%) [17]. We previously reported that in women without metabolic syndrome (MetS) at baseline, (neo)adjuvant chemotherapy adversely altered MetS components, body composition, and blood biomarkers related to inflammation and glucose metabolism after a 12–18-weeks of (neo)adjuvant chemotherapy [8]. However, the persistent effects of chemotherapy on MetS among breast cancer patients remain to be explored. Therefore, the purpose of our study was to examine the persistent (~ 4–5 years) effects of (neo)adjuvant chemotherapy on MetS components and related body composition and metabolic biomarkers among women diagnosed with early stage breast cancer patients with no pre-existing MetS.

## Methods

### Patients

We previously executed a prospective observational study that recruited women with newly diagnosed early stage breast cancer from the Medical Oncology clinics at the City of Hope [8]. This study was approved by the City of Hope Institutional Review Board. Informed consent was obtained from each participant prior to the baseline visit. A research nurse screened all new breast cancer patients for the following eligibility criteria: (1) women with newly diagnosed, stage I-III breast cancer; (2) age ≥ 18 years; (3) planned adjuvant chemotherapy following lumpectomy or mastectomy or planned neoadjuvant chemotherapy; (4) ability to sign informed consent. Participation in this study did not influence the treatment regimens women received. All patients received chemotherapy as determined by their treating oncologist.

Patients were ineligible if they had MetS at the time of chemotherapy. If a patient had any three of the five components, she was determined to have MetS (Table 1) [12]. No additional eligibility criteria were applied to this follow-up substudy. Further exclusion criteria included ≥ 10% weight loss within the previous 6 months or diagnosis of distant metastatic disease.

### Study measurements

All follow-up study measurements were performed on a single day during a follow-up medical oncology visit (within 4–5 years after the completion of chemotherapy) by trained research staff at the City of Hope.

Blood pressure: Blood pressure was measured at rest (patient seated for 5 min) using an automated BP device (Connex® ProBP™, Beaverton, OR) and was performed twice to ensure accuracy. The average of the two values was recorded and used to assess MetS.

Body composition: Height and weight measurements were used to calculate body mass index (BMI) in kg/m^2^. A portable hand-held bioelectrical impedance device (Omron®; Hoffman Estates, IL) was used to assess body composition (lean body mass, fat mass, and percent body fat). Waist and hip circumferences were measured using a measuring tape to determine the circumference of the waist (centered at the navel) and hip (centered on the greater trochanter), and used to calculate waist:hip ratio.

Biomarkers: A 12-h fasting blood sample was obtained for glucose, insulin, lipid profile [total cholesterol (TC), HDL-C, low-density lipoprotein cholesterol (LDL-C), and TRI], glycated hemoglobin (HbA1c), and C-reactive protein (CRP). Insulin resistance was calculated using the homeostatic model assessment (HOMA-IR): [fasting glucose (mg/dL) × fasting insulin (mg/dL)/405] [18]. Blood samples were analyzed at the City of Hope Clinical Pathology Laboratory. Lipids, glucose, insulin, and CRP assays were analyzed on the Vitros 4600 Analyzer (Ortho Clinical Diagnostics, Rochester, NY) using microslide technology. HbA1c was determined using high performance liquid chromatography (Diazyme, Poway, CA).

Number of MetS components: The average number of MetS components was determined by assessing the mean frequency at which each patient had a clinical feature of MetS out of five total features.

Physical activity assessment: History of physical activity [19] was assessed to capture fluctuating levels of physical activity throughout the lifetime including current levels, quantified as average minutes per week per year over each 5-year period from school-aged (ages 5–9, 10–14, 15–19 years) through adulthood (20–24, 25–29, 30–34, 35–39 years, etc., up to the time of diagnosis).

### Statistical analysis

Data were analyzed using SPSS version 18.0 (IBM, Armonk, New York). Standard methods were used to compute means, standard deviations (SDs), and frequencies. Paired sample t-tests were used to compare MetS, anthropometric, and metabolic biomarkers before initiating chemotherapy, after completing chemotherapy, and at follow-up. A comparison of means adjusting for age, race, type of chemotherapy, duration of chemotherapy, duration of follow-up, baseline BMI, and menopausal status was performed using repeated measures analysis of covariance (ANCOVA). Bonferroni’s multiple comparison post hoc tests were used to compare mean values. All statistical tests were conducted with two-sided alternative hypotheses, and p-values < 0.05 were considered statistically significant.

## Results

### Patient population

Over 4–5 years following chemotherapy, the 86 women from our baseline sample were recruited to participate in the follow-up visit. Reasons for lack of return to follow-up among those lost to follow-up (n = 58) were patient refused visit (56%), did not return calls/emails (34%), breast cancer recurrence (4%), metastatic disease (4%), deceased (2%), residing out of the state or country (1%). Table 2 presents the post-chemotherapy MetS variables for those lost to follow-up and those who returned for follow-up. There were no significant differences between these groups upon completion of chemotherapy. Baseline follow-up characteristics are displayed in Table 3. The mean age of the patients was 48.2 (± 10.1) years. The majority of patients were Caucasian (45%) or Hispanic (30%), nonsmoking (95%), employed (80%), and well-educated (90%). In general, the population was sedentary, averaging 47.2 (± 25.8) minutes of physical activity/week in the previous 12 months. The chemotherapy regimens included dose dense cyclophosphamide and doxorubicin followed by paclitaxel (42%), docetaxel and cyclophosphamide (36%), carboplatin and paclitaxel (9%), cyclophosphamide and doxorubicin (7%) or docetaxel, carboplatin, and trastuzumab (6%) patients. The average duration of chemotherapy was 15.3 weeks (± 2.7 weeks). The average duration of follow-up was 4.75 (± 0.65) years. Based on fasting blood glucose levels and confirmed from medical record abstraction, prediabetes (64%) and diabetes (21%) affected the majority of our sample.

**Table 2:** Post-chemotherapy MetS features among early stage breast cancer patients

Variable	Lost to follow-up^a^ (N = 58)	Follow-up^a^ (N = 28)	*P*^+^
Waist circumference, cm	91.3 (10.9)	90.7 (11.2)	P = 0.41
Blood pressure, mmHg			
Systolic	132 (15)	128 (27)	P = 0.32
Diastolic	92 (9)	90 (18)	P = 0.67
Fasting blood glucose, mg/dL	119.2 (27.8)	117.0 (37.0)	P = 0.44
Triglycerides, mg/dL	130.9 (55.6)	128.7 (58.9)	P = 0.69
HDL-C, mg/dL	53.9 (13.2)	50.6 (14.9)	P = 0.56

*HDL-C* high-density lipoprotein-cholesterol

^a^Mean (± SD)

^+^Between group comparison

**Table 3:** Baseline patient characteristics (N = 28)

Characteristic	Mean (± standard deviation)	N (%)
Age, years	54.1 (10.2)	
Race/ethnicity	Caucasian	13 (45)
	Asian	1 (7)
	Hispanic	9 (30)
	African American	2 (8)
	Other	3 (10)
Tobacco use	Never	14 (50)
	Current	1 (5)
	Past	13 (45)
Marital status	Married	23 (80)
	Single/divorced	4 (15)
	Widowed	1 (5)
Education level	High school or equivalent	10 (35)
	College or postgraduate degree	15 (55)
	Other	3 (10)
Employment status	Full-time	16 (60)
	Part-time	6 (20)
	Retired	6 (20)
Cancer stage	I	11 (40)
	II	14 (50)
	III	3 (10)
Surgery type	Mastectomy	13 (45)
	Lumpectomy	10 (35)
	N/A (neoadjuvant chemo)	5 (20)
Chemotherapy type	Doxorubicin/cyclophosphamide + paclitaxel	13 (42)
	Docetaxel/cyclophosphamide	11 (36)
	Carboplatin + paclitaxel	2 (9)
	Doxorubicin/cyclophosphamide	1 (7)
	Docetaxel/cyclophosphamide/trastuzumab	1 (6)
Diabetes status	Free from disease	4 (15)
	Pre-diabetic	18 (64)
	Diabetic	6 (21)

### Metabolic syndrome

Table 4 presents the individual components of MetS after follow-up. All component of MetS statistically significantly worsened at follow-up (p < 0.01) even when adjusted for age, race, type/duration of chemotherapy, duration of follow-up, and BMI. The percent changes observed ranged from 2.7% (waist circumference) to 17.9% (HDL-C) over the approximate 5-year duration of follow-up. The number of MetS components did not significantly change from post-chemotherapy to follow-up (p = 0.98).

**Table 4:** Follow-up changes in MetS after chemotherapy among early stage breast cancer patients

Variable	Pre-treatment^a^	Post-treatment^a^	Follow-up^a^	% Change^+^	*P*^+^
Waist circumference, cm	86.7 (12.9)	90.7 (11.2)	93.2 (12.2)	2.7	P < 0.01
Blood pressure, mmHg					
Systolic	122 (25)	128 (27)	133 (25)	3.8	P < 0.01
Diastolic	83 (13)	90 (18)	94 (20)	4.3	
Fasting blood glucose, mg/dL	97.2 (19.8)	117.0 (37.0)	125.2 (41.0)	6.6	P < 0.01
Triglycerides, mg/dL	108.7 (47.6)	128.7 (58.9)	144.6 (16.1)	11.0	P < 0.01
HDL-C, mg/dL	57.9 (12.0)	50.6 (14.9)	42.9 (16.8)	− 17.9	P < 0.01
# MetS components	1.0 (0.5)	4.0 (1.0)	4.0 (1.0)	0.0	P = 0.98

*HDL-C* high-density lipoprotein-cholesterol, *MetS* metabolic syndrome

^a^Mean (± SD)

^+^From post-treatment to follow-up

### Body composition

Follow-up body composition values are shown in Table 5. All measures of body composition were statistically significantly worsened at follow-up when compared to post-chemotherapy values (p < 0.01). The mean weight gain during follow-up was 6.1 kg (± 2.4 kg). Lean body mass was statistically significantly reduced at follow-up when compared to post-chemotherapy values (p < 0.01). Adverse changes in these anthropometric values remained statistically significant when adjusted for age, race, and type/duration of chemotherapy, and duration of follow-up.

**Table 5:** Follow-up changes in anthropometrics after following chemotherapy among early stage breast cancer patients

Variable	Pre-treatment^a^	Post-treatment^a^	Follow-up^a^	% Change^+^	*P*^+^
Height, cm	161.2 (7.4)	–	–	–	–
Weight, kg	69.2 (17.1)	74.7 (17.9)	80.8 (19.3)	8.2	P < 0.01
Body mass index, kg/m^2^	25.9 (6.3)	29.0 (7.0)	31.6 (7.9)	9.0	P < 0.01
Lean body mass, kg	45.8 (9.7)	47.1 (9.6)	43.5 (7.3)	2.9	P < 0.01
Fat mass, kg	23.6 (10.3)	27.6 (9.5)	31.5 (11.0)	12.3	P < 0.01
Body fat, %	33.1 (8.2)	36.0 (5.1)	39.3 (7.8)	8.3	P < 0.01
Hip circumference, cm	40.8 (4.3)	41.5 (7.4)	44.9 (8.8)	7.6	P < 0.01
Waist/hip ratio	0.84 (0.07)	1.1 (0.1)	1.4 (0.2)	21.4	P < 0.01

^a^Mean (± SD)

^+^From post-treatment to follow-up

### Metabolic biomarkers

Metabolic biomarkers are shown in Table 6. Notably, all biomarkers, TC, LDL-C, HOMA-IR, insulin, HbA1c, and CRP, were statistically significantly higher at follow-up when compared to post-chemotherapy values (p < 0.001), and remained statistically significantly elevated when adjusted for age, race, and type/duration of chemotherapy, and duration of follow-up. The percent changes in metabolic biomarkers observed varied from 10.6% (total cholesterol) to 24.6% (CRP) over the approximate 5-year duration of follow-up.

**Table 6:** Follow-up changes in metabolic biomarkers after chemotherapy among early stage breast cancer patients

Variable	Pre-treatment^a^	Post-treatment^a^	Follow-up^a^	% Change^+^	*P*^+^
Lipid profile					
Total cholesterol, mg/dL	185.5 (48.3)	201.9 (45.5)	225.8 (56.1)	10.6	< 0.01
LDL-C, mg/dL	100.5 (34.4)	111.1 (43.7)	134.5 (45.8)	17.4	< 0.01
Glucose metabolism					
Fasting insulin, mIU/mL	18.9 (21.8)	32.6 (17.3)	36.9 (15.4)	11.7	< 0.01
HOMA-IR	4.52 (1.1)	9.4 (1.5)	11.4 (1.3)	17.5	< 0.01
HbA1c, %	5.4 (0.4)	5.9 (0.6)	6.7 (0.8)	11.9	< 0.01
Inflammation					
CRP, mg/L	0.37 (0.36)	0.49 (0.21)	0.65 (0.2)	24.6	< 0.01

*LDL-C* low-density lipoprotein-cholesterol, *HOMA-IR* homeostatic model assessment-insulin resistance, *HbA1c* glycosylated hemoglobin, *CRP* C-reactive protein

^a^Mean (± SD)

^+^From post-treatment to follow-up

## Discussion

This is the first study, to date, to comprehensively address the persistent effect of (neo)adjuvant chemotherapy on MetS and associated biomarkers in breast cancer survivors. Previous studies with smaller sample sizes have reported MetS or its related components following completion of breast cancer treatments [20–22]. Within 5 years of treatment completion, MetS persisted in more than 85% of our study population. We also observed, these patients remain susceptible to additional health-related concerns, including diabetes, cardiovascular diseases, and cancer recurrence 4–5 years post-chemotherapy as noted by persistent weight gain, insulin resistance, and excess cholesterol. Our study highlights the potential long-term metabolic dysregulation in the years following completion of (neo)adjuvant chemotherapy. These finding emphasize the critical need for research efforts to focus on large randomized controlled trials of modifiable lifestyle factors to offset metabolic disturbances and improve prognosis, during and soon after treatment completion, and also in the years shortly thereafter [23].

Notably, women without a breast cancer diagnosis experience heightened risks of MetS and cardiovascular disease (CVD) particularly during the menopausal transition, which typically occurs in early to mid-50 years of age [24]. As the women in our sample are within this age range, it is prudent to examine trends in disease onset and progression. While there is a plethora of data to support adverse changes in body composition, lipid metabolism, insulin resistance, triglyceride levels as a result of menopause, the rate of onset and progression is highly variable with changes occurring within months to years before and following menopause [24]. Nonetheless, observational data reports a fourfold increase in CVD risk in the 10 years following natural menopause [25], thus in comparison our sample experienced an acceleration of said risk perhaps as a results of chemotherapy exposure.

Weight gain at 1 year post-treatment have been previously reported yet here we documented an 8% (~ 5 kg) increase in body weight at the 5-year follow-up. This surpasses the previously documented average of ~ 2–3 kg post-chemotherapy [2, 26]. A recent prospective study examining a cohort of breast cancer patients receiving chemotherapy reported that lower BMI at diagnosis predicted larger subsequent weight gain post-treatment [27], which perhaps explains the persistent weight gain in our sample whom had a normal pre-chemotherapy BMI. In our study, waist circumference significantly increased, averaging 93.2 cm at follow-up, indicating the worsening of central obesity during follow-up, which may drive the exacerbated metabolic disturbances observed in our population, heightening the risk of all-cause mortality [28].

An additional detrimental finding from our study is the enduring effect of chemotherapy on glucose metabolism. Fasting insulin increased by approximately 12% and HOMA-IR increased by approximately 17% over the 5-year study duration. Among early stage breast cancer patients, elevated levels of fasting insulin are associated with distant recurrence and mortality [29] and while elevated HOMA-IR scores are associated with reductions in breast cancer survival and all-cause survival [14]. In accordance with the increase of insulin resistance (HOMA-IR; 17.5%), is the progressive increase in HbA1c (11.9%). Previously we reported the no significant alteration in HbA1c following chemotherapy yet 5-years post-treatment we observed a significant change, perhaps due to the longer duration of follow-up.

Among the metabolic biomarkers, CRP levels worsened drastically at an increase of approximately 25%. As CRP is indicator of inflammation and potential mediator of the obesity-cancer association, it is highly plausible that the continual worsening is attributable to obesity [30] and insulin resistance [31] observed in our sample. In accordance with the present study, CRP was moderately-severely elevated (5.1 ± 5.3 mg/dL) in 91% of overweight breast cancer survivors (n = 42) who were using adjuvant hormone therapy [32]. Collectively, CRP remains an important metabolic biomarker post-treatment, highly prone to worsening over time that should be closely monitored throughout survivorship, in addition to insulin resistance, especially among obese, insulin resistant breast cancer survivors.

There are several plausible mechanisms that could elucidate the observed persistent metabolic deterioration approximately 5 years after chemotherapy in our study. Weight gain remains a critical motivating factor as our participants experienced approximately 8% gain in body weight, 12% gain in fat mass, and 9% increase in BMI. Thus, when classifying by BMI, an overweight sample of survivors noted immediately following chemotherapy was reclassified as obese 5 years later. Waist circumference, an indirect assessment of visceral adipose tissue, increased in our sample by approximately 3%, thus potentially promoting the development of inflammation [33] and insulin resistance [34, 35]. In addition, our sample experienced a 3% decline in lean body mass at follow-up, thus presenting with lower lean body mass compared to pre-chemotherapy. Due to the positive role of skeletal muscle tissue in the regulation of glucose metabolism [36], this slight wasting may further contribute to the persistent worsening of insulin resistance.

Given the profound metabolic disturbance observed over the treatment trajectory, it is of importance to consider non-pharmacologic approaches to offset said comorbidities. Exercise is an effective non-pharmacologic strategy to mitigate cancer-related treatment side effects [37, 38]. Combined aerobic and resistance exercise has shown to have beneficial outcomes on metabolic disturbances [37, 39], however, few studies have focused on the early survivorship period (≤ 6 months post-treatment) in order to prevent said disturbances from worsening, metabolically unhealthy and obese women, or tested a combined exercise program. We previously reported that a 16-week combined aerobic and resistance exercise intervention led to significant improvements in metabolic syndrome, sarcopenic obesity, circulating biomarkers, muscle strength, and psychosocial health among metabolically unhealthy, obese, sedentary breast cancer survivors who had completed cytotoxic treatment in the previous 6 months [40]. Despite these promising results, it remains to be determined as to whether exercise can elicit sustained benefits on all-cause mortality among breast cancer survivors.

We acknowledge limitations of our study. First, we did not assess dietary intake at the follow-up assessment because participants perceived it as overly burdensome at the earlier time points. Second, although our initial sample size was the largest to date to study MetS during chemotherapy, only approximately 33% of our original sample participated in the follow-up measures, representing a smaller cohort of women previously treated for breast cancer. Third, our sample did not include a large percentage of Asian or African American patients. Fourth, there is potential bias in our sample as the patients who did complete follow-up may have been healthier given their willingness to return to clinic, and thus our data underrepresents the metabolic deterioration of our entire sample obtained at the pre-chemotherapy assessment. Finally, our study is void of a control group to assess whether the patients would have experienced these persistent changes in the absence of chemotherapy.

## Conclusion

In conclusion, breast cancer patients who underwent (neo)adjuvant chemotherapy experienced persistent metabolic dysregulation as noted by the exacerbation of MetS, body composition, and glucose metabolism from immediately following chemotherapy to approximately 5 years following the completion of chemotherapy. Our findings suggest the need to strategize lifestyle interventions to counterbalance these negative metabolic effects or to introduce as preventive measures prior to, during, and following treatments for breast cancer.

## Data Availability

The datasets used and/or analyzed during the current study are available from the corresponding author on reasonable request.

## Abbreviations

ANCOVA: Analysis of covariance
BF: Percent body fat
BG: Blood glucose
BMI: Body mass index
BP: Blood pressure
BW: Body weight
CRP: C-reactive protein
FM: Fat mass
HbA1c: Hemoglobin A1c (glycated hemoglobin)
HDL-c: High density lipoprotein-cholesterol
HOMA-IR: Homeostatic model-insulin resistance
LDL-C: Low density lipoprotein-cholesterol
MetS: Metabolic syndrome
SD: Standard deviation
TC: Total cholesterol
TRI: Triglycerides
WC: Waist circumference
